# Idalopirdine, a selective 5-HT_6_ receptor antagonist, reduces food intake and body weight in a model of excessive eating

**DOI:** 10.1007/s11011-017-0175-1

**Published:** 2018-01-03

**Authors:** Magdalena Kotańska, Klaudia Lustyk, Adam Bucki, Monika Marcinkowska, Joanna Śniecikowska, Marcin Kołaczkowski

**Affiliations:** 1 Department of Pharmacodynamics, Jagiellonian University, Medical College, 9 Medyczna Street, 30-688 Kraków, Poland; 2 Department of Medicinal Chemistry, Jagiellonian University, Medical College, 9 Medyczna Street, 30-688 Kraków, Poland; 3 Adamed Ltd., Pieńków 149, 05-152 Czosnów, Poland

**Keywords:** Idalopirdine, LuAE58054, 5-HT_6_ receptor antagonist, Anorectic activity, Excessive eating model

## Abstract

Obesity, from early childhood onwards, is a common societal problem. The overconsumption of sweet, salty and high-fat products are the main factors that cause excessive weight gain. It is therefore necessary to search for new drugs that affect satiety centers and reduce the sense of hunger and caloric intake. It has been suggested that the blockade of 5-HT_6_ receptors may reduce food intake, and since idalopirdine is a clinically tested, selective 5HT_6_ receptor antagonist, it was chosen to be examined in animal models of obesity. The activity of idalopirdine was measured in the rat model of excessive eating. Animals were on a high caloric diet that consisted of milk chocolate with nuts, cheese, salted peanuts and condensed milk. During a four-week experiment, the rats had constant access to standard feed and water ad libitum. Idalopirdine was administered intraperitoneally at a dose 5 mg/kg b.w./day. To establish whether idalopirdine would effectively suppress the rebound hyperphagia that accompanies refeeding, it was administered after a 20 h food deprivation period. Pica behavior was evaluated after the administration of idalopirdine to confirm that the suppression of food intake was not caused by visceral illness. The effect of the four-week treatment with idalopirdine on the amount of peritoneal adipose tissue, and on lipid and carbohydrate profiles in rats was also examined. The statistical significance was calculated using the one-way ANOVA post-hoc Tukey Multiple Comparison Test or the two-way ANOVA post-hoc Bonferroni Multiple Comparison Test. Idalopirdine significantly reduced caloric intake and prevented the development of obesity in tested animals. Rats, that received idalopirdine, had a smaller amount of adipose tissue in the peritoneum as well as lower glucose, triglyceride and cholesterol levels in comparison to the control group. Moreover, an anorectic action was not caused by abnormalities of the gastrointestinal tract, such as nausea. The obtained results indicate that idalopirdine reduces caloric intake and could be considered for further tests as a potential treatment of obesity.

## Introduction

The epidemic of obesity is mostly responsible for the increasing prevalence of the metabolic syndrome (Rutigliano et al. 2017) and thus effective treatment of obesity still remains an important aim of public health worldwide. Obesity, from early childhood onwards, is a common societal problem (Zeyda and Stulnig 2009). A very large group of children aged 10 are either overweight, or already obese (WHO 2015). The overconsumption of sweet, salty and high-fat products are the main factors that cause obesity (WHO 2015). It is therefore necessary to search for new drugs that affect satiety centers and reduce the sense of hunger and caloric intake.

Idalopirdine is a potent and selective 5-HT_6_ receptor antagonist (Ki = 0.83 nM) (Arnt et al. 2010) developed by Lundbeck and Otsuka Pharmaceuticals, as an adjunctive therapy for the treatment of cognitive deficits associated with Alzheimer’s disease. Mørk et al. (2017) indicated that idalopirdine increased cortical levels of dopamine and noradrenaline. As of October 2013, it is in phase III clinical trials (Galimberti and Scarpini 2015; Wilkinson et al. 2014). Idalopirdine has been proven to be safe and well tolerated at single doses of up to 360 mg in clinical pharmacology studies (Ellen Schmidt, Lundbeck, Valby, Denmark, personal communication) and at a daily dose of 30 mg thrice during 24 weeks of randomized, double-blind, placebo-controlled phase II trials (LADDER) (Wilkinson et al. 2014). Besides the promising procognitive effects, the 5-HT_6_ receptor blockade has been implicated in the reduction of food intake, body weight, as well as visceral adiposity and insulin resistance (Heal et al. 2008). 5-HT_6_ receptor antagonists block the serotonin-dependent activation of γ-aminobutyric acid (GABA) neurons, which results in a reduction of inhibitory effects of GABA on pro-opiomelanocortin neurons in the arcuate nucleus, with subsequent inhibition of hunger signal induction (Sargent and Henderson 2011). Since idalopirdine is the most advanced selective 5-HT_6_ antagonist in development, it was selected by us for studying the effects on food intake. We have recently shown that idalopirdine significantly decreased food intake and the amount of peritoneal fat as well as reduced the level of plasma triglycerides in obese animals (Dudek et al. 2015). As a follow up, in the present study, we tested the effect of idalopirdine on food intake and body weight in the model of excessive eating (Western diet) in rats, including the effect on the rebound hyperphagia. The performed studies could constitute a premise for further preclinical and clinical evaluation of idalopirdine as a potential treatment of obesity.

## Materials and methods

### Animals

The experiments were carried out on male Wistar rats weighting between 200 g and 230 g. The animals were housed in plastic cages (3 rats per cage) at a constant room temperature of 22 ± 2 °C, with 12:12 h light/dark cycle. Water and food were available ad libitum. Each control and experimental group consisted of six to eight animals. All experiments were conducted in accordance with the Guide to the Care and Use of Experimental Animals and were approved by the Local Ethics Committee for Experiments on Aminals of the Jagiellonian University in Krakow (2013 and 2015, Poland; approval numbers 136/2013 and 258/2015).

### The influence of idalopirdine on body weight and food and water intake in rats fed with palatable diet (western-style diet) and normal diet

In order to determine the anorectic activity of idalopirdine, its effect on caloric and water intake in the model of excessive eating was assessed (Kotańska et al. 2017). Two groups of six rats were fed during four weeks with a diet consisting of milk chocolate with nuts, cheese, salted peanuts and 7% condensed milk. Animals had access to standard food (Labofeed B, Morawski Manufacturer Feed, Poland) and water ad libitum.

The palatable control group received intraperitoneally a vehicle (5% 2-hydroxypropyl-beta-cyclodextrin) whereas idalopirdine palatable group received 5 mg/kg b.w. of idalopirdine in 5% 2-hydroxypropyl-beta-cyclodextrin.

Palatable diet contained: 100 g peanuts – 614 kcal; 100 ml condensed milk – 131 kcal; 100 g milk chocolate with hazelnuts – 195 kcal; 100 g Greek cheese – 270 kcal.

Two other groups of rats were on a standard diet (100 g feed - 280 kcal)*.* A vehicle (5% HP-beta-cyclodextrin) was administered intraperitoneally to the control group, while idalopirdine (5 mg/kg b.w./day) in 5% 2-hydroxypropyl-beta-cyclodextrin was given to idalopirdine group.

**Fig. 1:**
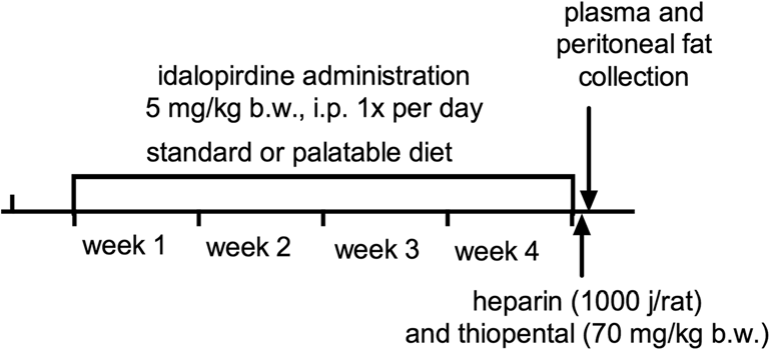
A schematic diagram of chronic administration of idalopirdine in model of excessive eating . Idalopirdine (5 mg/kg b.w.) was administered intrapiritoneally to rats for 28 consecutive days. Control groups received 5% 2-hydroxypropyl-beta-cyclodextrin

The consumption of food and water was evaluated three times per week and body weight of animals was measured daily, immediately before administration of substances. On the 29th day of the experiment, 20 min after intraperitoneal administration of heparin (1000 j/rat) and thiopental (70 mg/kg b.w.), plasma was collected from the left carotid artery and peritoneal fat was weighed (Fig. 1).

### Influence on acute feed consumption after 20-h feed deprivation in rats (model of hyperphagia after refeeding)

**Fig. 2:**
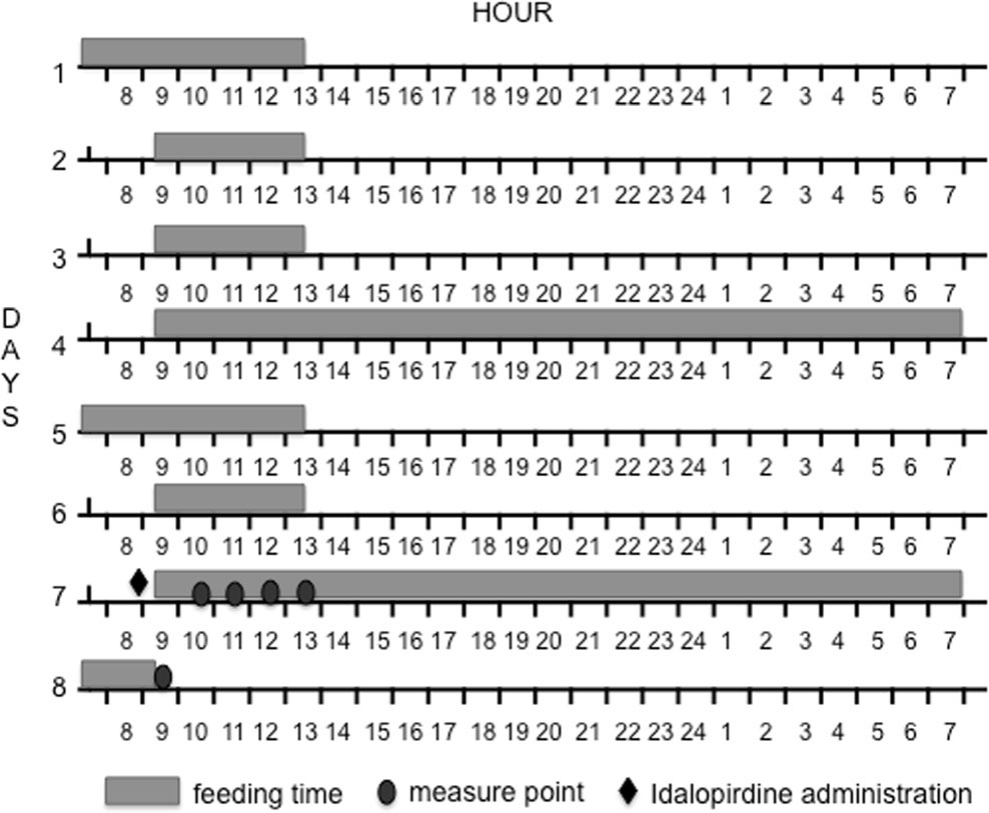
A schematic diagram of feeding scheme, compound administration and measure points in model of influence on acute feed intake after feed deprivation period model

To establish whether idalopirdine would effectively suppress the rebound hyperphagia that accompanies refeeding, the rats were taught that they would have access to food for only 4 h during the day. That procedure was implemented on the first, second and third day of the experiment, while the animals were given their feed at 9:30 AM and had it removed at 1:30 PM. On the 4th day, the feed was served at 9:30 AM and remained in the cage until 1:30 PM of the 5th day. On the 6th day, the feed was served at 9:30 AM and remained until 1:30 PM. On the 7th day of the experiment, idalopirdine was administered intraperitoneally at a dose of 5 mg/kg b.w., 30 min before serving feed (9.00 AM). The control group received only the vehicle. The amount of consumed feed was evaluated every hour, starting at 10:30 AM until 1:30 PM and then after 24 h (Fig. 2).

### Influence on visceral illness via measurement of kaolin intake (Pica behavior)

To exclude the possibility that the suppression of food intake by idalopirdine was caused by visceral illness, pica behavior was evaluated. The method was based on the works by Takeda et al. (1993) and Yamamoto et al. (2002). The experiment lasted five days. In addition to free access to feed, animals had free access to the white kaolin. For the first few days, the animals were accustomed to the presence of kaolin in their cages. On the 4th day, either idalopirdine (5 mg/kg b.w.), a vehicle (negative control group), or a solution of CuSO_4_ (6 mg/kg b.w. - 1/3 LD_50_; LD_50_ = 18 mg/kg for a rat at this route of administration; positive control group) was administered intraperitoneally. The amount of consumed food, water and kaolin was determined after 24 h. Moreover animals were weighed before the administration of the compounds and 24 h after.

### Influence on lipid profile and glucose level in plasma

To determine the lipid profile and glucose level in plasma, standard enzymatic and spectrophotometric tests (Biomaxima S.A. Lublin, Poland) were carried out. The substrate was decomposed with enzymes suitable to the relevant product, which was then converted to a colored compound. The coloration was proportional to the concentration. The absorbance was measured at a wavelength of 500 (glucose, triglycerides, total cholesterol).

### Data analysis and statistical procedures

Statistical calculations were carried out with GraphPad Prism 6 software. The presented results are the means ± S.E.M. The statistical significance was calculated using the one-way analysis of variance (ANOVA) post-hoc Tukey Multiple Comparison Test or the two-way ANOVA post-hoc Bonferroni Multiple Comparison Test. Differences considered were statistically significant at: *, ^ *p* ≤ 0.05, **,^^ *p* ≤ 0.01, ***, ^^^ *p* ≤ 0.001.

### Drugs, chemical reagents and other materials

Heparin was provided by Polfa Warszawa S.A. (Warsaw, Poland), while thiopental sodium by Sandoz International (Stryków, Poland) and 5% 2-hydroxypropyl-beta-cyclodextrin by Sigma-Aldrich, USA.

Idalopirdine was synthesized in the Department of Medicinal Chemistry, Faculty of Pharmacy, Jagiellonian University Medical College, Kraków, Poland according to the procedure described previously (Pasternak and Szymonifka 2009). The structure was confirmed by proton and carbon nuclear magnetic resonance (^1^HNMR, ^13^CNMR) obtained through a Varian BB 200 spectrometer using tetramethylsilane (TMS) (0.00 ppm) in chloroform-d1. The purity was established by ultra-performance liquid chromatography-mass spectrometry (UPLC-MS) analysis at >98%. The UPLC/MS system consisted of a Waters Acquity UPLC, coupled to a Waters single quadrupole detector (SQD) mass spectrometer. Chromatographic separations were carried out using an Acquity UPLC Ethylene Bridged Hybrid (BEH) C18 column, 2.1 × 100 mm and 1.7 μm particle size. The column was maintained at 60 °C and eluted under the following gradient conditions: 4 min, a linear gradient from 80 to 0.1% of eluent A at a flow rate of 0.5 ml/min. Eluent A: water/formic acid (0.02%, *v*/v) and eluent B: methanol – acidic gradient. Eluent A: water/formic acid/ammonia solution (0.01%/0.1%, v/v/v) and Eluent B: methanol – alkaline gradient.

## Results

### Obesity induced with palatable diet

**Fig. 3:**
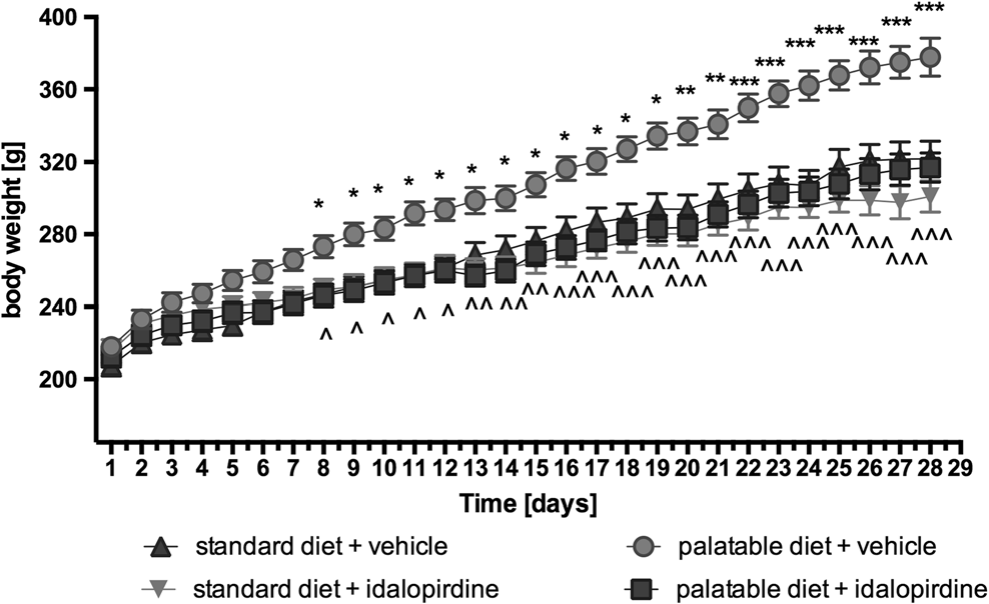
Effect of long-term administration of the 5-HT_6_ receptor antagonist, idalopirdine, on body weight in male Wistar rats in the model of excessive eating . Results are means ± SEM, *n* = 6. Multiple comparisons of the vehicle-treated control group against the vehicle-treated palatable control group (*) or idalopirdine-treated palatable group against the vehicle-treated palatable control group (^) were calculated using the two-way ANOVA, post-hoc Benferroni test. Significant differences are denoted by *, ^ *p* < 0.05; **, ^^ *p* < 0.01; ***, ^^^ *p* < 0.001

Animals fed with the palatable diet indicated significantly higher weight gain than the group fed with the standard feed (from day 8 of the experiment). On the 8th day, the difference was 6.51%, whereas on the 28th day it was 14.82%. Throughout the experiment, rats from the control group fed with standard feed put on 55% of their initial weight, while rats from the control group fed with a palatable feed had a weight gain of 73.38%. Results are shown in Fig. 3.

### Effect of idalopirdine on body weight, caloric and water intake

The animals fed with palatable feed and treated with idalopirdine showed significantly less weight gain than rats from the control group consuming a preferential feed. From the 8th day of the experiment, a statistically significant difference in body weight between the groups was observed (on day 8–5.02% and on day 28–16.09%). Most importantly, the body weight of rats treated with idalopirdine that had access to preferential feed did not differ significantly from the body weight of rats from the control group fed with a standard feed. No effect on body weight was noticed in animals treated with idalopirdine and those who consumed standard feed. Results are shown in Fig. 3.

**Fig. 4:**
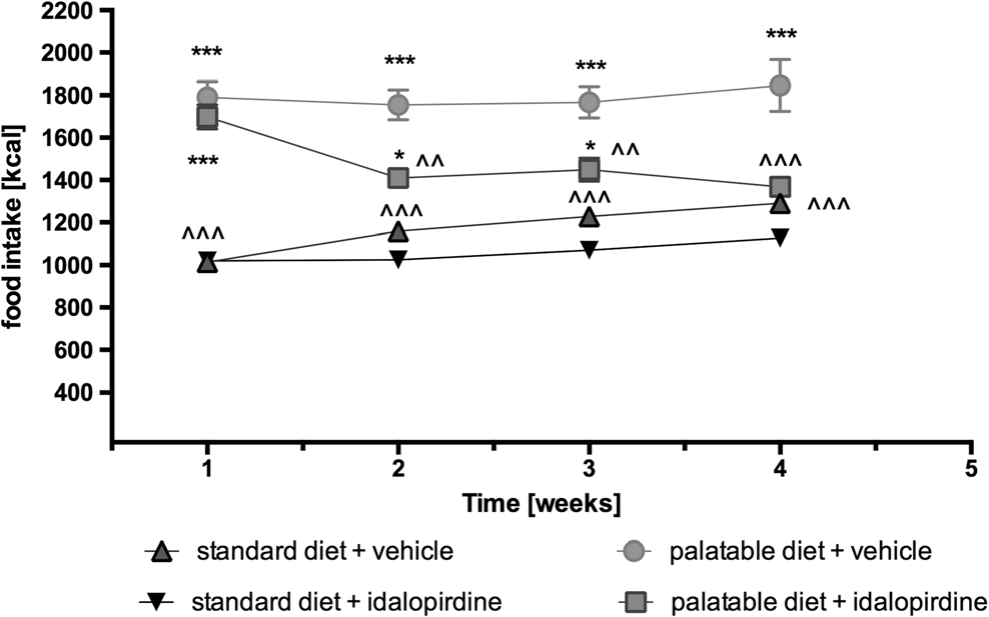
Effect of long-term administration of the 5-HT_6_ receptor antagonist, idalopirdine, on food intake in male Wistar rats in the model of excessive eating . Results are means ± SEM, data for three animals reared together. Multiple comparisons against the vehicle-treated control group (*) or against the vehicle-treated palatable control group (^) were calculated using the two-way ANOVA, post-hoc Bonferroni test. Significant differences are denoted by *, ^ *p* < 0.05, **, ^^ *p* < 0.01, ***, ^^^ *p* < 0.001

Idalopirdine administered intraperitoneally at the dose of 5 mg/kg b.w. significantly reduced the amount of consumed calories by the animals on a palatable diet in comparison to the ones of the control group. No differences were observed in caloric intake from the group that had access only to the standard feed. Results are shown in Fig. 4.

**Fig. 5:**
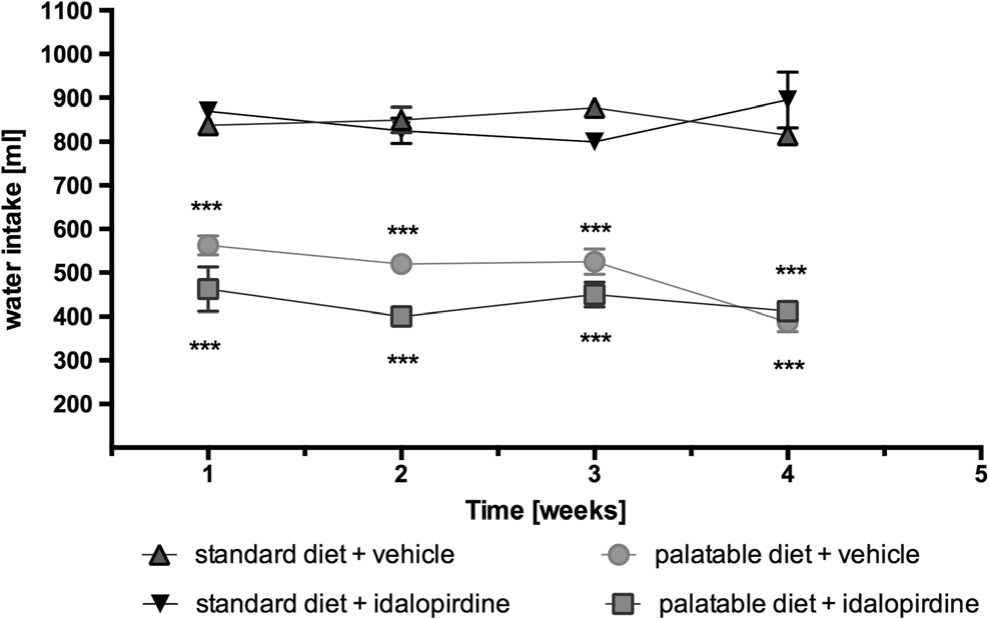
Effect of long-term administration of the 5-HT_6_ receptor antagonist, idalopirdine, on water intake in male Wistar rats in the model of excessive eating . Results are means ± SEM, data for three animals reared together. Multiple comparisons against the vehicle-treated palatable control group (*) were calculated using the two-way ANOVA, post-hoc Bonferroni test. Significant differences are denoted by * p < 0.05, ** *p* < 0.01, *** *p* < 0.001

There were no significant differences in water intake by animals between groups fed with the same feed. On the other hand, significantly less water consumption was noticed in the case of animals fed with preferential feed. Results are shown in Fig. 5.

### Influence on acute feed consumption after 20-h feed deprivation in rats

**Fig. 6:**
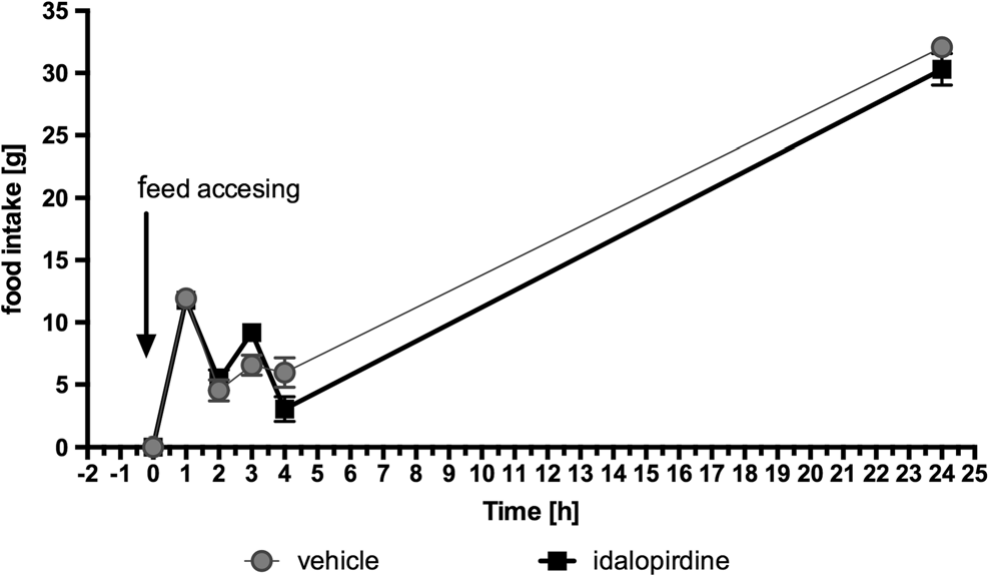
Effect of one administration of the 5-HT_6_ receptor antagonist, idalopirdine, on food intake in male Wistar rats in the model of refeeding hyperphagia . Results are means ± SEM, data for two animals reared together, *n* = 6. Multiple comparisons against the vehicle-treated control group were calculated using the two-way ANOVA, post-hoc Bonferroni test

After 20 h of fasting, idalopirdine was administered 30 min before eating. For the first two hours, the amount of consumed feed was comparable to that consumed by rats from the control group. On the third hour, slightly more feed was consumed and later slightly less. The results were non-significant. Results are shown in Fig. 6.

### Effects on visceral illness via measurement of kaolin intake (Pica behavior)

**Fig. 7:**
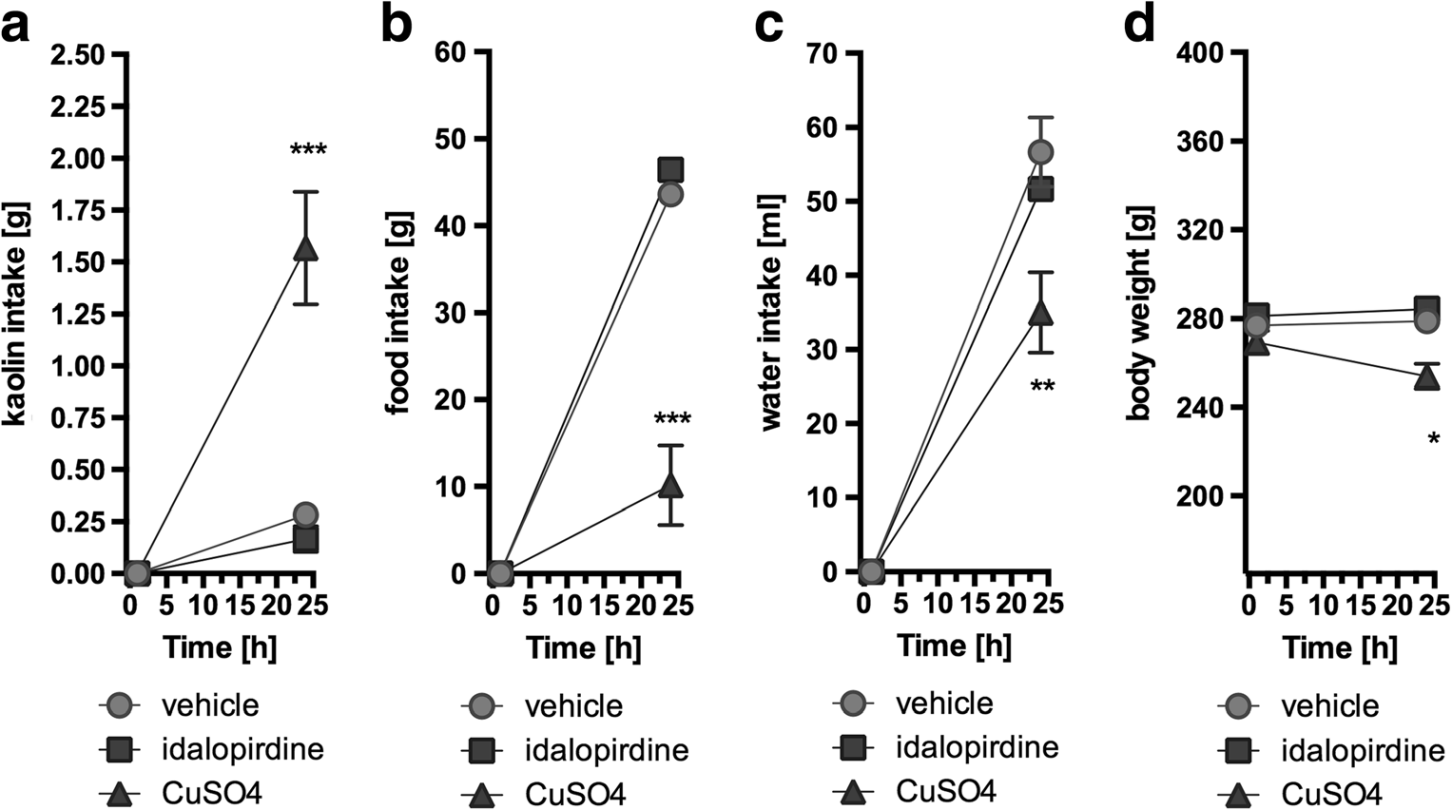
Effect of one administration of the 5-HT_6_ receptor antagonist, idalopirdine, on kaolin intake (a), food intake (b), water intake (c) and body weight (d) in male Wistar rats in the model of pica behavior . Results are means ± SEM, data for two animals reared together, *n* = 6. Multiple comparisons against the vehicle-treated control group (*) were calculated using the two-way ANOVA, post-hoc Bonferroni test. Significant differences are denoted by * *p* < 0.05, ** *p* < 0.01, *** p < 0.001

Animals that received idalopirdine intraperitoneally at dose of 5 mg/kg b.w. did not consume more kaolin compared to the control group that received only the vehicle. The second control group, that was given CuSO_4_, had a significantly greater intake of kaolin and a significantly lower intake of feed and water. This was accompanied by body weight decrease. Results are shown in Fig. 7.

### Influence of the 4-week chronic treatment with idalopirdine on the amount of peritoneal adipose tissue and lipid and carbohydrate profiles in rats

**Fig. 8:**
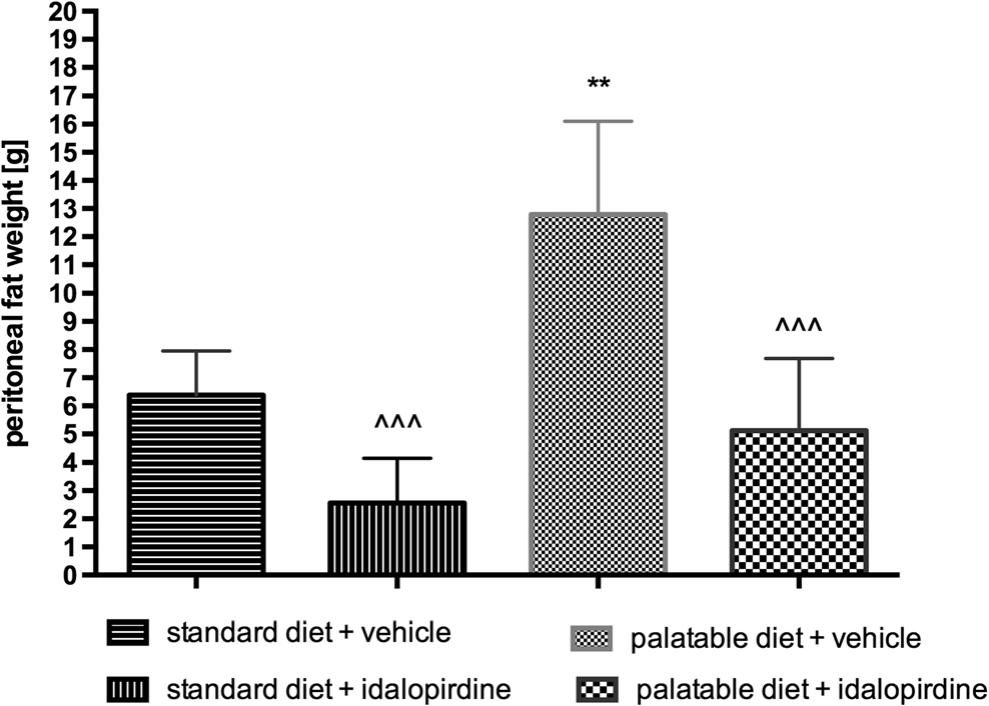
Weight of peritoneal fat after long-term administration of the 5-HT_6_ receptor antagonist – idalopirdine in male Wistar rats in the model of excessive eating . Results are means ± SEM, n = 6. Multiple comparisons against the vehicle-treated control group (*) or against the vehicle-treated palatable control group (^) were calculated using the two-way ANOVA, post-hoc Bonferroni test. Significant differences are denoted by ** p < 0.01, ^^^ p < 0.001

Animals consuming palatable feed had statistically significantly more fat in peritonea. The group, which received the tested compound, had a significantly lower amount of fat in the peritonea compared to the obese rats. The results are shown in Fig. 8.

**Fig. 9:**
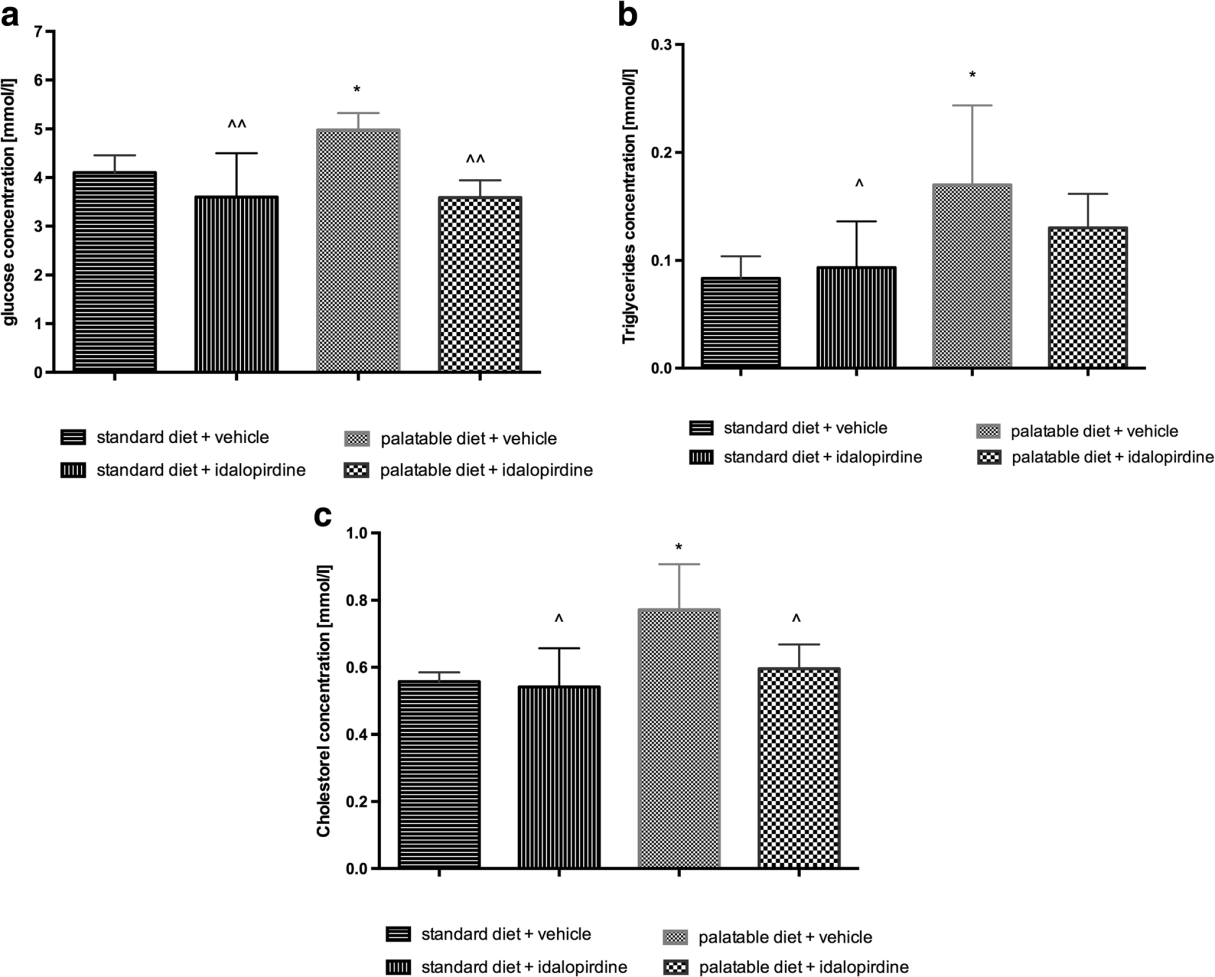
Effects of long-term administration of the 5-HT_6_ receptor antagonist, idalopirdine, on plasma: glucose (a), triglyceride (b), and total cholesterol (c) levels in male Wistar rats in the model of excessive eating . Results are means ± SEM, n = 6. Concentrations in plasma: mmol/l. Comparisons against the vehicle-treated control group (*) or against the vehicle-treated palatable control group (^)were calculated using the one-way ANOVA, post-hoc Tukey test. Significant differences are denoted by *, ^ p < 0.05, ^^ p < 0.01

Moreover, the level of glucose, triglyceride and cholesterol in blood was higher in rats on the palatable diet than on the standard diet. Rats treated for four weeks with idalopirdine and fed with a palatable diet had a significantly lower level of glucose, triglycerides and cholesterol in plasma in comparison to the control group fed with the palatable diet. The results are shown in Fig. 9.

## Discussion

Idalopirdine is a selective 5-HT_6_ receptor antagonist, which is being tested in phase III clinical trials as a potential treatment of cognitive deficits in Alzheimer’s disease (Galimberti and Scarpini 2015; Wilkinson et al. 2014). Numerous reports have indicated that the 5-HT_6_ receptor antagonists, from different chemical classes, reduce food intake and could be considered as potential treatment of obesity (Heal et al. 2008). However, it is highly unlikely, that a drug candidate for Alzheimer’s disease would be clinically tested as an anti-obesity treatment, without previous preclinical evidence suggesting such an activity. To the best of our knowledge, so far there has been no preclinical evaluation of idalopiridine in the animal model of excessive eating, although its mechanism of action indicates potential influence on the reduction of food intake. Our goal was therefore to provide a basis for further, extended preclinical and clinical testing of idalopirdine in that indication.

As a result of the present study, we found that idalopirdine significantly reduced caloric intake and prevented the development of obesity in tested animals.

In the model of excessive eating from the 8th day of experiment, the animals that consumed the palatable diet weighed significantly more than the animals that ate only the standard feed. During the experiment, the difference in weight increased significantly, highlighting how quickly and effectively obesity can develop in the presence of an unlimited source of highly caloric products, which animals prefer to eat. An increase in the amount of body fat, blood glucose, triglycerides and cholesterol was also observed. Animals fed with preferential feed and treated with idalopirdine consumed significantly less calories than the control group fed with preferential feed and gained less weight than the untreated animals. Similar results were previously obtained in a model of obesity induced through the administration of high-fat feed. It was observed that obese animals ate fewer calories and lost weight after receiving idalopirdine (Dudek et al. 2015).

In the normally fed, non-obese animals, idalopiridine did not disrupt body weight gain, indicating no risk of such side effects for normal weight conditions. A decrease in the body weight of animals, below normal values as well as a reduction in the consumption of a normal diet could be considered as an unacceptable side effect, however, in the present study this was not the case. This observation is in line with clinical results, indicating no weight loss in non-obese patients treated with idalopirdine (Wilkinson et al. 2014).

Furthermore, administration of idalopirdine in animals that had access to excess calories, decreased the amount of fat in the peritoneum, as well as significantly lowered plasma glucose, triglyceride and cholesterol levels, in comparison to the control group fed with similar feed. The observed changes in biochemical parameters were evidently associated with a decrease in excessive caloric intake.

Food consumption in rats and mice can also be decreased through various factors including stress, sickness, sedation or drug-induced toxicity and not only through the enhancement of satiety or other specific mechanisms (Vickers et al. 2011). Dudek et al. (2015) showcased that idalopirdine did not significantly affect spontaneous activity of animals that remain permanently under standard conditions in cages. Therefore, it should be assumed that the reduction of food intake observed in this study were not due to sedation. Moreover, if that were the case, a decrease in food intake in the group fed with standard feed would also be observed.

Some drugs may reduce food intake by producing gastrointestinal malaise, which is difficult to detect in animal behavior. Rats and mice lack the emetic response, which distinguishes them from humans. However, the persistent eating of inert substances by rodents can be used to evaluate illness-response behavior analogous to vomiting in other species; this behavior is called pica (Vickers et al. 2011; Takeda et al. 1993; Yamamoto et al. 2002). In the present study, animals also had access to kaolin clay. After being administered idalopirdine, rats did not consume the clay, which proved that disorders of the gastrointestinal tract, such as visceral irritation or nausea, did not occur. The consumption of kaolin clay, food and water did not differ significantly in the group administered with idalopirdine, compared to the negative control group. On the other hand, CuSO_4_, used as a positive control in this test, extensively reduced the food and water intake and significantly increased the consumed amount of kaolin clay, indicating the stomach upset.

The effect of a single dose of the tested compound on the amount of ingested food is often used as a screening of potential food intake reduction (Vickers et al. 2011). Such activity can be observed after a single administration of clinically active substances such as: d-fenfluramine, sibutramine, rimonabant or lorcaserin (Jackson et al. 1997; Colombo et al. 1998; Neill and Cooper 1989; Smith et al. 2008). Animals used in such an experiment are not required to be obese (Vickers et al. 2011). The present study included an experiment that determined the influence of idalopirdine on rebound hyperphagia which accompanied refeeding. The results were established after a single administration of idalopirdine to the fasted animals, which were taught that the feed would be provided only for a limited time. Idalopirdine showed the influence on the amount of food intake, but it was not statistically significant. Although the differences in food consumption that occurred in the 3rd, 4th and 24th hour of feed availability were not statistically significant, they confirm that the tested compound affects nutritional behavior. It is possible that the 5HT_6_ receptor antagonists are incapable of inducing statistically significant effect in such an experiment after a single administration, just as they actually do not affect the amount of food intake after a single administration, without application of the model of controlled fasting (Heal et al. 2008). However, due to the special feeding scheme required prior to administration of the compound, it is not feasible to carry out the same experiment after repeated administration.

## Conclusion

In the present study we showed that idalopiridne, a selective 5-HT_6_ receptor antagonist, tested in phase III clinical trials as a cognitive enhancer in Alzheimer’s disease, is able to reduce caloric intake and prevent the development of obesity in the model of excessive eating. The effect was proven not to be related to gastrointestinal malaise or sedation, testifying for its specificity. Together with the previously shown anorectic effects of idalopirdine in obese animals (Dudek et al. 2015), the present findings constitute an important prerequisite for further preclinical and clinical investigation of idalopirdine as potential anti-obesity agent.

## Abbreviations

5HT_6_ receptor: 5-hydroxytryptamine receptors
ANOVA: analysis of variance
BEH: Ethylene Bridged Hybrid
^13^CNMR: carbon nuclear magnetic resonance
GABA: γ-Aminobutyric acid
^1^HNMR: proton nuclear magnetic resonance
SEM: standard errors of the mean
SQD: single quadrupole detector
TMS: tetramethylsilane
UPLC: ultra performance liquid chromatography
UPLC-MS: ultra performance liquid chromatography – mass spectrometry
